# Removal of Hexavalent Chromium in Portland Cement Using Ground Granulated Blast-Furnace Slag Powder

**DOI:** 10.3390/ma11010011

**Published:** 2017-12-22

**Authors:** Sungchul Bae, Fumino Hikaru, Manabu Kanematsu, Chiaki Yoshizawa, Takafumi Noguchi, Youngsang Yu, Juyoung Ha

**Affiliations:** 1 Department of Architectural Engineering, Hanyang University, Seoul 04763, Korea; 2 Faculty of Science and Technology, Tokyo University of Science, 2641 Yamazaki, Noda, Chiba 278-8510, Japan; waraimaker@gmail.com (F.H.); manabu@rs.noda.tus.ac.jp (M.K.); 3 Research Institute of Science and Technology, Nihon University College of Science and Technology, Chiyoda, Tokyo 101-8308, Japan; chiaki.yoshizawa@orsc.co.jp; 4 Department of Architecture, Graduate school of Engineering, The University of Tokyo, Bunkyo, Tokyo 113-8654, Japan; noguchi@bme.arch.t.u-tokyo.ac.jp; 5 Advanced Light Source, Lawrence Berkeley National Laboratory, Berkeley, CA 94720, USA; ysyu@lbl.gov; 6 School of Environmental and Sustainability Sciences, Kean University, Union County, NJ 07083, USA; juyoung@gmail.com

**Keywords:** hexavalent chromium, reduction, immobilization, slag, Portland cement

## Abstract

Using ground granulated blast-furnace slag (GGBS) under different alkaline conditions, we studied the mechanisms and extents of Cr(VI) reduction and sorption and compared them to reactions with Portland cement (PC). We also investigated the effects of mixing PC/GGBS ratios on Cr(VI) dissolution after carbonating the substrates. We observed a complete sorption and reduction of Cr(VI) to Cr(III) in a GGBS-in-Ca(OH)_2_ solution (pH > ~12.5) after 10 h, whereas in distilled water (pH = ~11.5) GGBS exhibited only marginal sorption and reduction (20%). Cr reactions with dissolved ions in supernatants derived from GGBS indicated that the anions dissolved from GGBS act as a reducing agent for Cr(VI) in a Ca(OH)_2_ solution. Soft X-ray absorption microscopy identified a partial reduction of Cr(VI) to Cr(III) on the GGBS surface. The carbonation of pure PC paste substantially increased the amount of dissolved Cr(VI) in a solution phase whereas a 5 wt % replacement of PC with GGBS significantly reduced the amount of dissolved Cr(VI). We concluded that in the mixed paste during the early curing stage GGBS reduced a significant fraction of Cr(VI) to Cr(III) and that the Cr(III) adsorbed in the GGBS-PC mixture’s hydration products does not readily dissolve, even under carbonation conditions.

## 1. Introduction

Hexavalent chromium [Cr(VI)] is found in concrete as chromate, which results from the oxidation process of trivalent chromium [Cr(III)] during Portland cement (PC) clinker production [1,2]. Chromium (Cr) can exist in several different oxidative states and its physical and chemical properties, such as solubility, toxicity, and sorption affinity, vary with environmental conditions. For example, Cr(III) is likely to precipitate in alkaline solutions, whereas Cr(VI) is amphoteric with a concentration-dependent solubility [3]. Cr(VI) is highly mobile but very toxic under aerobic conditions [1,4,5]. Cr(III) has a strong affinity to soils and other substrates; while it is less soluble and toxic than Cr(VI), its potential for oxidation to Cr(VI) should be carefully examined in order to fully understand Cr transport and distribution in the environment. Therefore, examining Cr(VI) dissolution, sorption, and reduction in PC clinker is of the utmost important for environmental concerns and for the long-term sustainability of PC materials in construction materials [3,6].

In the past, stabilization and solidification (s/s) have been practiced on immobilized PC-associated Cr(VI) [7,8,9]. Stabilization involves a reductive transformation of Cr(VI) to Cr(III) and a subsequent chemical transformation into a highly insoluble compound, such as calcium chromate [10,11]. Solidification involves the co-precipitation of Cr(VI) with dissolved PC hydration products during hydration reactions [10]. Calcium silicate hydrate (C-S-H), which is the primary hydration and binding product in PC-based materials, contains most of the microporosity, resulting in a large specific surface area that provides physical sorption and co-precipitation sites for heavy metal ions like Cr(VI). It should also be noted that Zamorani et al. [12] observed co-precipitated phase of CaCrO_4_ when Cr(VI) reacted with C-S-H, suggesting that molecular-level surface reactions are important in determining the reaction rates and pathways when hydrated products of PC-based materials react with Cr(VI) [3]. Ettringite (3CaO·Al_2_O_3_·3CaSO_4_·32H_2_O) has also been identified as one of PC’s hydration products and, with its highly reactive and needle-like column structure, has been proposed as a surface site for Cr(VI) immobilization via anion substitution, where CrO_4_^2−^ is exchanged for SO_4_^2−^ [13,14]. Another study identified that reductive reactions occur during the curing phases and that the resulting Cr(III) also replaces Al(III) in the ettringite crystalline structure [15]. Ionic substitutions in ettringite were also observed to be Cr(VI)–ettringite and Cr(III)–ettringite phases that further limit the mobility of Cr [8,16]. Cr(VI) can be substituted for SO_4_^2−^ in the interlayer of calcium aluminate monosulfate (hereafter referred to as monosulfate, 3CaO·Al_2_O_3_·CaSO_4_·12H_2_O) [16]. However, the pH stability range of reactions for Cr(VI)–ettringite and Cr(VI)-hydration products of PC is very narrow and varies significantly [16,17].

The alkalinity of the PC-based matrix is important for decreasing the dissolution of Cr ions [3,11]. However, the carbonation process neutralizes the alkaline conditions and increases the Cr(VI) solubility for PC pastes [18]. A decrease in the pH conditions due to carbonation causes the decomposition of Cr(VI)-bearing hydration products in cementitious materials, which leads to more dissolved aqueous Cr(VI) [19].

Ground granulated blast-furnace slag (GGBS) is a well-known by-product of the pig-iron manufacturing process, and up to 60–70% of GGBS is often incorporated into concrete in order to recycle industrial waste materials [20]. GGBS is capable of immobilizing Cr(VI) via a reductive process: dissolved S^2−^ ions from GGBS transform Cr(VI) into Cr(III) [3]. On the contrary, Macias et al. [18] observed an increased dissolution of Cr(VI) in GGBS-PC pastes due to the Cr(VI) that was released into the cement materials’ pore solution. The effect of GGBS on Cr(VI) reduction and immobilization has not yet been quantitatively studied, and the exact mechanism of GGBS remains unclear.

The aim of this study is to elucidate the mechanism of GGBS on Cr(VI) speciation under different alkaline levels. Ca(OH)_2_ was utilized in order to maintain a high alkaline condition for the activation of GGBS. We also studied GGBS’s capacity to reduce and immobilize Cr(VI), comparing it to that of pure PC. We performed quantitative determinations of the Cr(VI) using a UV-visible spectrophotometer. The dissolved cations and anions in Cr(VI)’s reductive transformation in the solution phase were quantified under different alkaline conditions using ion chromatography. The phases for each sample that was made to react with different Cr(VI) concentrations were identified using X-ray diffraction (XRD). Synchrotron-based X-ray microscopy and spectroscopy were used to identify the detailed chemical speciation of Cr that had reacted with the Ca(OH)_2_-activated GGBS particles. Finally, an accelerated carbonation test was performed to examine the effects of carbonation on Cr(VI) leaching from a GGBS-PC paste.

## 2. Experimental Methods

### 2.1. Material and Sample Descriptions

The chemical compositions of PC and GGBS were determined using X-ray fluorescence (XRF, Bruker, Karlsruhe, Germany) (Table 1). Research-grade PC was used instead of commercial PC to minimize any possible impurities and their subsequent interference with our experimental results.

Table 2 shows the mix proportions and experimental measurements for the different samples. Four different series of experimental samples were prepared, and they are denoted as S1, S2, S3, and S4. In the S1 samples, we examined Cr(VI) reduction and immobilization using PC or GGBS. A Cr(VI) solution (0.5 mg/L) was prepared by dissolving potassium dichromate (K_2_Cr_2_O_7_) in distilled water. The GGBS or PC powder (40 g) was added into the Cr(VI) solutions, and the suspensions were agitated at 20 °C in 60% relative humidity (RH) conditions. The supernatant of the S1 samples was filtered, and the concentration of the Cr(VI) remaining in the filtered solution was determined. The changes in pH of the S1 suspensions were measured via a hand-held pH meter (D-52, Horiba, Tokyo, Japan).

S2 samples were prepared in order to verify what effects the dissolved ions from PC or GGBS had on Cr(VI) reduction in the solution phase. The PC or GGBS powder (48 g) was mixed with distilled water (2.4 L), and the substrates were equilibrated in distilled water for 24 h to ensure the dissolution of the substrates. After 24 h, the supernatant was filtered, and 1.2 mg of K_2_Cr_2_O_7_ was added to the filtered solutions containing the dissolved ions from the paste samples. Changes to the initial Cr(VI) concentration (0.5 mg/L) was measured at 0, 1, 3, 6, 12, and 24 h in the reacted solutions to identify the extent of the reductive reaction that was due to the dissolved ions from the substrates in the solutions.

The S3 experiments series aimed to determine the Cr(VI) sorption capacity of GGBS. Different amounts of Cr(VI) ranging from 0.5 to 100 mg/L were added to a GGBS suspension. After agitating the suspension, the remaining Cr(VI) concentration was determined at 120 and 150 h. Lastly, the S4 series was used to determine the different chemical species of the dissolved free ions from the GGBS samples under different alkaline condition. Ion chromatography (Dionex ICS-2000, Sunnyvale, CA, USA), using suppressed conductivity detection, was performed to quantify the ions that were dissolved in S4 samples. The Cr(VI) concentration in S4 samples was measured using a UV-visible spectrophotometer (DR 5000™, Hach, London, ON, Canada) and the EPA 7196A method with 1,5-diphenylcarbazide (Cr(VI) Detection limit, Max: 1.0 mg/L, Min: 0.03 mg/L). All the solutions were filtered through a 0.45-μm membrane filter, and the alkaline conditions of the solutions were maintained using pure Ca(OH)_2_.

### 2.2. Accelerated Carbonation and Tank Leaching Test

GGBS-PC pastes (ø50 × 100 mm) were made at GGBS replacement ratios of 0, 5, 10, and 50 wt %. The water to total solid (GGBS + PC) ratio (w/s) of the pastes was maintained at 0.6 for all the samples. The total initial Cr(VI) concentration was at 0.5 mg/L per 100 g of paste. During the first 24 h of curing, plastic sheets covered the exposed surfaces of the specimen in the mold. The specimens were demolded after 24 h and were cured for an additional 28 days at 20 °C in 60% RH conditions. For accelerated carbonation, the final sample was finely grounded and placed in a carbonation chamber at 20 °C with 5% CO_2_ and 60% RH for 28 days. After the carbonation period was completed, the specimens were kept in an ethanol suspension and stored in a vacuum drying chamber until further analysis. The hydration products were identified both before and after 28 days of carbonation. After carbonation, the remaining Cr(VI) concentration in the samples was measured according to Regulation No. 49 (The Ministry of Land, Infrastructure and Transport, Japan).

### 2.3. Phase Identification

The raw material phases, the hydration products in the filtered solids derived from the S1 and S3 suspensions, and the accelerated carbonation test specimens before and after carbonation were all identified via powder XRD using monochromatized CuKα radiation (λ = 1.54 Å, at a voltage of 30 kV, a current of 10 mA, in the range of 2θ = 5°–75°, D2 Phaser, Bruker, Karlsruhe, Germany). The residues collected after filtering were dried and used for the XRD analysis. Prior to the sample preparation, both the carbonation of the raw materials and the purity of Ca(OH)_2_, which was used as a reference compound, were checked using XRD. Although great care was taken to avoid carbonation of the pure Ca(OH)_2_, very small peaks of CaCO_3_ were seen in the XRD pattern, and the amount of CaCO_3_ was quantified as ~2 wt % via Rietveld refinement using the Topas software (version 4.3, Bruker, Karlsruhe, Germany). This result may be due to the unavoidable carbonation of pure Ca(OH)_2_ during the XRD measurements.

### 2.4. Scanning Transmission X-ray Microscopy (STXM)

Spatial correlations between the chemical compositions and their oxidation states were mapped by a combination of scanning transmission X-ray microscopy (STXM) and X-ray absorption spectroscopy (XAS) across Cr *L*_2,3_-edges (560–600 eV) and the O *K*-edge (525–560 eV), which allows us to image distributions of the chemical species with spectral sensitivity from very small spots (~35 nm). STXM measurements were performed at the bending magnet beamline (5.3.2.1) at the Advanced Light Source (ALS) in the Lawrence Berkeley National Laboratory, Berkeley, CA, USA [21]. To obtain transparency in the soft X-ray region, the diluted mixtures (dried leachate of GGBS-CH-S3 and 10 mg/L of added Cr(VI)) were drop-casted with isopropanol onto a 50-nm-thick Si_3_N_4_ membrane (Silson Inc., Warwickshire, England). STXM measurements utilized a 25-nm outer-width Fresnel zone plate for focusing the X-ray. A raster scanned onto 8 × 8 μm^2^ field-of-views with a square scan grid of 35 nm steps. Image spectra (i.e., repeated images at different energies), whose finest energy step was 0.1 eV near the absorption resonance, were recorded by a phosphor photomultiplier tube (PMT). The intensity transmitted through the samples (*I*) was converted into the optical density (OD) by taking the log ratio of the incident (*I*_0_) and transmitted (*I*) intensities (Equation (1)):

(1) $$\mathrm{OD}=-\ln\left(\frac{I}{I_{0}}\right)=\sigma \cdot t=\mu \cdot \rho \cdot t,$$

where *I*_0_: incident X-ray photon intensity, *t*: specimen thickness, *μ*: mass absorption coefficient, and *ρ*: density of the specimen.

To overcome spectral distortions resulting from the acquisition time gap between the *I*_0_ and *I* measurements, the incident beam intensity was simultaneously measured through an open area of the sample. After image registration by a phase-correlation algorithm, the XAS spectra of each pixel as a function of energy were corrected. All data processing was carried out using the Axis2000 software (version 8.3, McMaster Univ., Hamilton, ON, Canada) [22]. Further details regarding applications of STXM to the study of cementitious systems can be found elsewhere [23,24,25,26].

## 3. Results and Discussion

### 3.1. Cr(VI) Removal Using PC and GGBS-PC Samples

**Figure 1:**
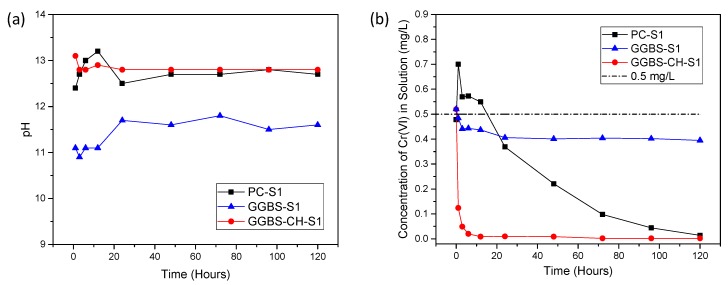
Time profiles of (**a**) the pH values of S1 samples’ suspensions and (**b**) the Cr(VI) concentrations in the S1 samples’ filtered solutions measured from 0 to 120 h.

Figure 1a shows the time profiles of the pH values for the suspensions retrieved from the S1 samples over 120 h. The pH value for PC-S1 increased from 12.7 to 13.2 in 12 h and decreased to 12.5. This is in good agreement with PC paste, which has a water to binder ratio (W/B) of 0.3 [27]. No significant changes in pH were observed afterwards. On the other hand, the pH value for GGBS-S1 increased from 11.1 to 11.7 in 24 h and remained in the 11.6–11.8 range until 120 h had passed. When compared to PC-S1 and GGBS-S1, GGBS-CH-S1, to which Ca(OH)_2_ was added, exhibited comparatively small changes in pH within the first 20 h. It had a constant value of 12.7 until 120 h had passed. Past studies indicate that GGBS’s activation is strongly dependent on pH values [28,29]. Higher pHs lead to improved GGBS activation, with a value higher than 11.5 leading to an effective activation [30]. Therefore, alkaline conditions for GGBS activation can be maintained by adding Ca(OH)_2_ (GGBS-CH-S1). The effect of alkalinity on GGBS activation was further confirmed in the XRD results (see Section 3.4).

Figure 1b depicts the Cr(VI) concentration of the supernatants ([Cr(VI)]_supernatants_) retrieved from the S1 samples as a function of time. Within 1 h, [Cr(VI)]_supernatants_ increased from 0.50 to 0.70 mg/L for PC samples. Following this, [Cr(VI)]_supernatants_ gradually decreased over 120 h to below the detection limit (0.03 mg/L). The initial increase in the Cr(VI) concentration is likely due to the initial dissolution of the cement clinkers in the PC samples. In the GGBS-S1 without Ca(OH)_2_, the [Cr(VI)]_supernatants_ decreased from 0.5 to 0.4 mg/L within 5 h and remained relatively constant at 0.40 mg/L over 120 h. In contrast, a rapid decrease was observed for the GGBS-CH-S1 in Ca(OH)_2_ solution, with its the [Cr(VI)]_supernatants_ reaching 0 mg/L within 24 h. This is an interesting observation because increasing the pH condition is expected to increase [Cr(VI)]_supernatants_. Instead, we observed a [Cr(VI)]_supernatants_ decrease in the presence of the GGBS paste. Our results suggest that GGBS has an impact on Cr(VI) reaction pathways in Ca(OH)_2_ conditions and that alkali-activated GGBS can remove and immobilize Cr(VI) more than the untreated GGBS in distilled water can.

### 3.2. Cr(VI) Reduction via Dissolved Ions from GGBS

**Figure 2:**
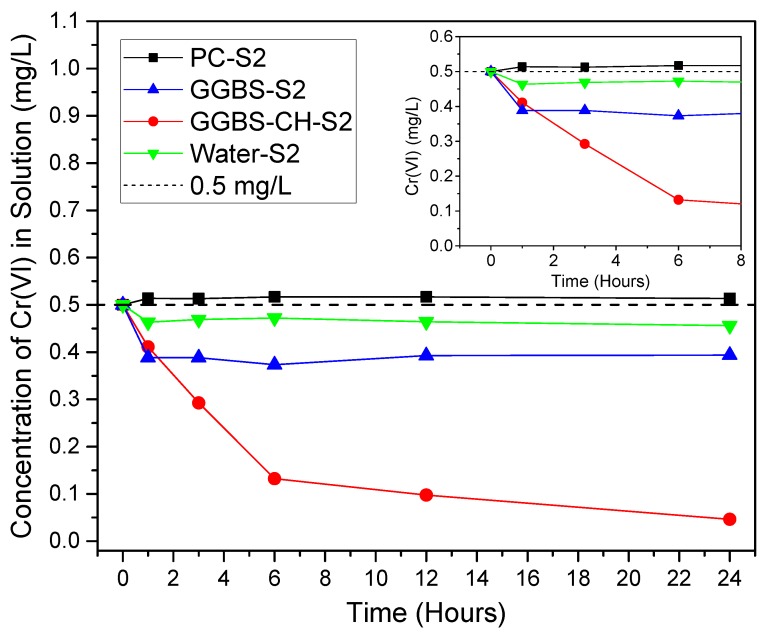
Time profile of the Cr(VI) concentration in the S2 specimens’ solutions from 0 to 24 h.

Figure 2 shows the evolution of the Cr(VI) concentration in the filtered solutions ([Cr(VI)]_aqueous_) of the S2 samples as a function of time. [Cr(VI)]_aqueous_ decreases when it reacts with dissolved ions from PC and GGBS. Water-S2, the control sample, is a mixture composed of distilled water and K_2_Cr_2_O_7_ mixed with a pre-determined Cr(VI) concentration of 0.5 mg/L, brought to reaction. The initial [Cr(VI)]_aqueous_ for all the samples was 0.5 mg/L. During the first hour, a slight increase in [Cr(VI)]_aqueous_ for the PC-S2 sample (~0.52 mg/L), when compared to Water-S2 (~0.47 mg/L), was likely due to Cr(VI) dissolving from the cement clinker, which we also observed in Figure 1b because PC inherently contains a small amount of trace Cr(VI).

The [Cr(VI)]_aqueous_ in GGBS-S2 decreased to 0.38 within 1 h before reaching a constant concentration. On the contrary, due to the presence of Ca(OH)_2_, GGBS-CH-S2 showed a significant and continuous Cr(VI) reduction over 6 h before finally reaching 0.05 mg/L at 24 h, which suggests an almost a complete removal of Cr(VI) from the solution. Based on the results, we attributed the reduced amount of Cr(VI) in the solution to the solution chemistry between the dissolved ions in the GGBS-CH-S2 sample with Cr(VI). In contrast, our experimental data indicated that the dissolved ions from the PC did not reduce the Cr(VI). Our results suggested that the significant Cr(VI) concentration decrease for the GGBS in Ca(OH)_2_ solution was primarily due to the reducing reaction between the dissolved ions from the GGBS and Cr(VI).

**Figure 3:**
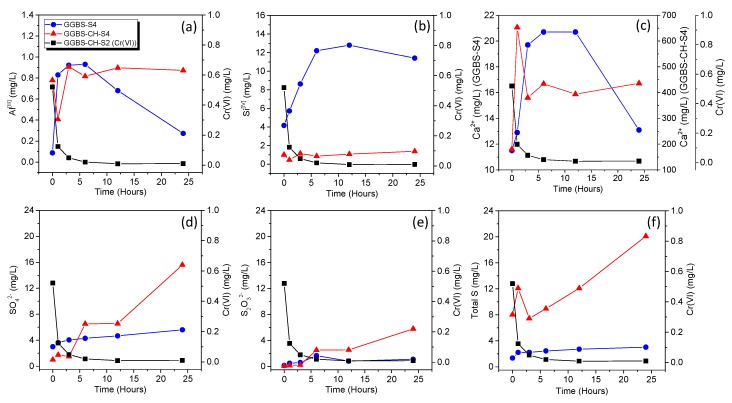
Time profiles for the ion dissolution from GGBS in distilled water (GGBS-S4) and GGBS in Ca(OH)_2_ solutions (GGBS-CH-S4) for 0 to 24 h. The Cr(VI) concentration in the GGBS-CH-S2 solution (Figure 2) is presented in the same plot. The maximum value of the y-axis was adjusted to the concentration value of each ion. (**a**) Al^[III]^; (**b**) Si^[IV]^; (**c**) Ca^2+^; (**d**) S_2_O_3_^2−^; (**e**) SO_4_^2−^; and (**f**) total S.

Figure 3 shows the evolution of the concentrations of the dissolved ions from the GGBS in water (GGBS-S4) and GGBS in Ca(OH)_2_ solution (GGBS-CH-S4) within 25 h. In both GGBS-S4 and GGBS-CH-S4 samples, the concentrations of Al^[III]^ were relatively lower (lower than 1 mg/L) than those recorded for the other cations. For GGBS-S4, the three cations (Al^[III]^, Si^[IV]^, and Ca^2+^) showed similar dissolution trends. We presumed that the concentration changes in those cations resulted from the slow dissolution of GGBS in water. The Si^[IV]^ concentration in GGBS-CH-S4 was very low due to the rapid consumption of Si in GGBS’s alkali-activation process.

The initial Ca^2+^ concentration in GGBS-CH-S4 (~200 mg/L) was much higher than for GGBS-S4 (~12 mg/L) because of the added Ca(OH)_2_. After 1 h, the Ca^2+^ concentration in GGBS-CH-S4 increased significantly to ~650 mg/L but then decreased to 400 mg/L at 3 h. After this, we recorded no changes in the Ca^2+^ concentration in GGBS-CH-S4. The initial rapid increase and the subsequent decrease of the Ca^2+^ concentration represent, respectively, the dissolution of Ca^2+^ from the GGBS and the ion’s consumption via the formation of alkali-activation products. The constant Ca^2+^ concentration that we recorded after 3 h resulted from the balance between the GGBS dissolution process on the one hand and the Ca^2+^ consumption via the precipitation of alkali-activation products on the other.

The S_2_O_3_^2−^ and SO_4_^2−^ concentrations were measured to represent the anions’ dissolution form the GGBS-S4 and GGBS-CH-S4 samples. In GGBS-S4, a relatively small amount of anions were dissolved from GGBS, and this amount was constant for 25 h. Conversely, in the GGBS-CH-S4 sample, the S_2_O_3_^2−^ and SO_4_^2−^ dissolution rapidly increased after 7 h. Most importantly, in contrast to the S_2_O_3_^2−^ (0~0.5 mg/L) and SO_4_^2−^ (1~2 mg/L) concentrations, we observed that a significantly greater amount of total sulfur (S) (7~13 mg/L) was dissolved within 5 h (the rapid Cr(VI) removal duration for GGBS-CH-S2, in Figure 2) in GGBS-CH-S4 samples than in GGBS-S4. Previous XAS study found that the S in GGBS exists mostly in the form of S^2−^ with a minor amount of SO_4_^2−^ [31]. Our results therefore suggested that GGBS samples in Ca(OH)_2_ solution the significant Cr(VI)-concentration decrease in the solution phase was primarily caused by the fact that the reducing reaction between the dissolved ions (S^2−^) acted as a reducing agent for the Cr(VI) in the solution, as has previously been found by others [3,11]. We concluded that the dissolved ions from the GGBS activated in Ca(OH)_2_ solution play a critical role in the reductive transformation of Cr(VI) into Cr(III) in solution.

### 3.3. Cr(VI) Adsorption onto GGBS-PC Samples

**Figure 4:**
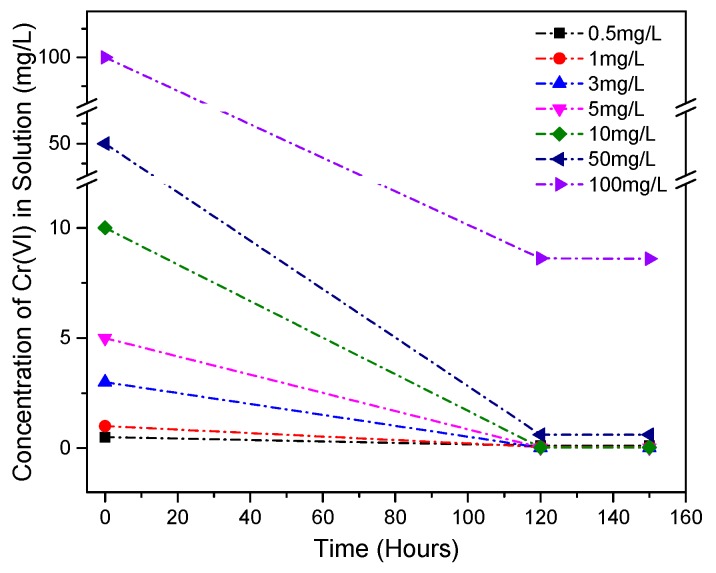
A comparison of the Cr(VI) concentrations in the S3 specimens’ solutions at 0, 120, and 150 h of reaction.

We measured the changes in the Cr(VI) concentration for the S3 samples at 0, 120, and 150 h in order to further evaluate the capacity of GGBS to immobilize Cr(VI) in a Ca(OH)_2_ solution (Figure 4). For each specimen, the initial Cr(VI) concentrations varied from 0.5 to 100 mg/L. After 120 h of agitation, we observed no trace of Cr(VI) in GGBS-CH-S3 that had a Cr(VI) concentration of up to 10 mg/L, which indicated the complete removal of Cr(VI) from that solution. The GGBS specimen containing 50 mg/L of Cr(VI) had approximately 0.7 mg/L of Cr(VI) after 120 h. The Cr(VI) concentration in the specimen containing 100 mg/L of Cr(VI) was reduced to 8.6 mg/L. Furthermore, for all samples, we observed no further change in the Cr(VI) concentrations even after 150 h. It is possible that all GGBS’s sorption sites for Cr(VI) are saturated at this concentration, which would explain why no further removal of Cr(VI) from the solution was observed. It is also possible that the saturation of GGBS’s surface sites hinders any further reduction of Cr(VI) to Cr(III). Based on there being no further changes in the Cr concentrations after over 150 h, a pseudo-equilibrium condition was attained under our experimental condition. We do not have the experimental data to measure activated complexes at the transition state for the overall Cr transformation reaction mechanisms, and as a consequence of this we cannot calculate the pseudo-equilibrium constant for the reactions. Further detailed work is needed to confirm the exact mechanism at play during surface site saturation, along with its impact on Cr(VI) uptake and reduction by GGBS. Additionally, GGBS in distilled water (GGBS-S1) exhibited a limited capacity for Cr(VI) removal within 120 h: 0.025 mg/L per gram of GGBS. Related to this result is the fact that GGBS’s capacity to immobilize and stabilize Cr(VI) in Ca(OH)_2_ solution was particularly large: approximately 2.3 mg/L per gram of GGBS. This result indicates that the alkaline conditions improve the dissolution of GGBS. This results in the enhancement of the GGBS’s capacity to remove Cr(VI), in addition to its potential to do so in distilled water conditions. All the reaction products of the experimental samples were further analyzed using XRD in order to examine the possible surface precipitate phase.

### 3.4. XRD Characterization of the Reacted Phases

**Figure 5:**
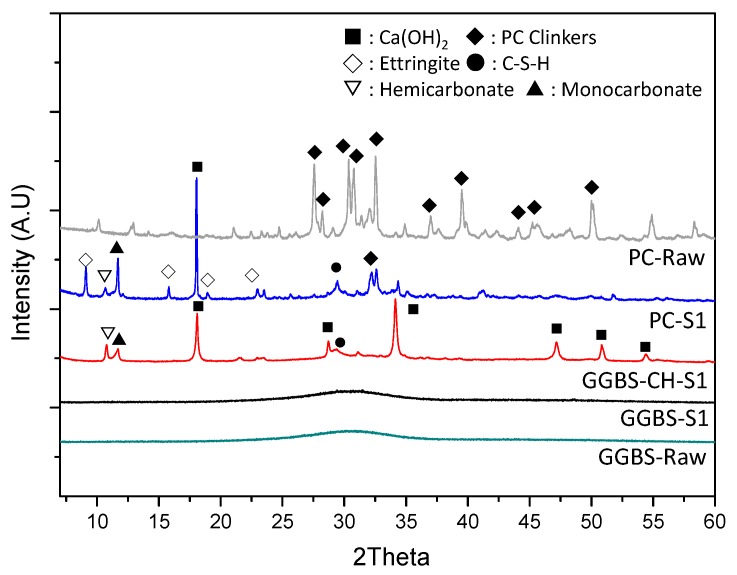
XRD patterns for raw materials (PC and GGBS), PC-S1, GGBS-CH-S1, and GGBS-S1.

Figure 5 shows the diffraction patterns of raw materials and of the S1 samples, measured using XRD. We recorded no diffraction peaks for K_2_Cr_2_O_7_ in S1 specimens, indicating the complete dissolution of K_2_Cr_2_O_7_. PC-S1 exhibited very strong crystalline peaks for the Ca(OH)_2_ formed from the PC hydration, but such crystalline peaks were absent in GGBS-S1’s XRD pattern, indicating that here GGBS was mostly amorphous and had not been effectively activated under a pH of circa −11.5 (Figure 1a). In the XRD patterns of PC-S1 and GGBS-CH-S1 samples, we observed a formation of calcium aluminate hemicarbonate (hereafter referred to as hemicarbonate, 2Ca_4_[Al(OH)_6_]_2_(CO_3_)_0.5_OH·5.5H_2_O) and calcium aluminate monocarbonate (hereafter referred to as monocarbonate, 3CaO·Al_2_O_3_·CaCO_3_·10.68H_2_O) while only the PC-S1 sample formed ettringite.

**Figure 6:**
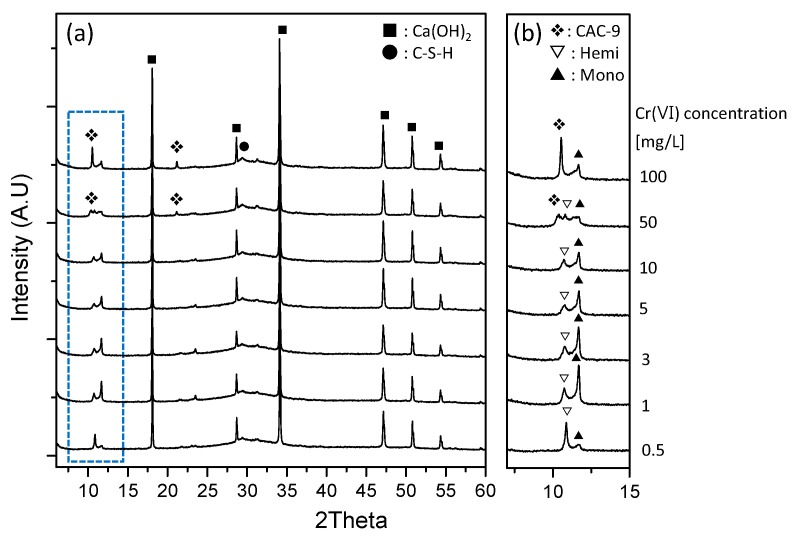
(**a**) XRD patterns of GGBS-CH-S3 in solutions containing 0.5 to 100 mg/L of Cr(VI) and (**b**) the magnified XRD patterns in the range of 9 to 15 degrees.

Figure 6a shows the XRD patterns for GGBS-CH-S3 with Cr(VI) concentrations ranging between 0.5 and 100 mg/L. Regardless of the initial Cr(VI) concentration, all the specimens exhibited very strong crystalline peaks due to the Ca(OH)_2_. In specimens with a Cr(VI) concentration of 0.5 mg/L (Figure 6b), hemicarbonate and monocarbonate formed, as observed in GGBS-CH-S1. No ettringite or monosulfate phases were found in any of the S3 specimens. In contrast, in the GGBS-CH with 50 and 100 mg/L of Cr(VI), the hemi- or monocarbonate XRD peak intensity was very low. The addition of 100 mg/L of Cr(VI) encouraged the formation of calcium aluminum Cr oxide hydrates ((Ca_4_Al_2_O_3_(CrO_4_)·9H_2_O), CAC-9), which suggests that the amount of Cr(VI) influences the formation of GGBS products under Ca(OH)_2_ activation. Previous studies have shown that Cr(VI) can be immobilized by pure PC hydrates, such as ettringite, mono-, and hemicarbonate [11,18]. Additionally, high-surface area hydration by-products, such as C-S-H from PC hydration reactions, can remove Cr(VI) from solutions via physical adsorption [3]. Based on our findings, i.e., the rapid Cr(VI) reduction and immobilization through GGBS under alkaline conditions maintained by Ca(OH)_2_, we conclude that the by-products produced by the alkali-activated GGBS can contribute to both the immobilization of Cr(VI) ions via the complexation with dissolved anions resulting from the GGBS hydration reaction, and the Cr(VI) reduction to Cr(III).

### 3.5. Chromium Speciation via STXM

In exhibiting different Cr oxidation states, Cr(III) and Cr(VI) can be readily resolved by comparing the sample spectra with suitable reference spectra that are taken at the Cr *L*_2,3_-edge. This is because near edge X-ray absorption fine structure (NEXAFS) is highly sensitive to both the oxidation states and the geometry of Cr’s first coordination shell [32].

**Figure 7:**
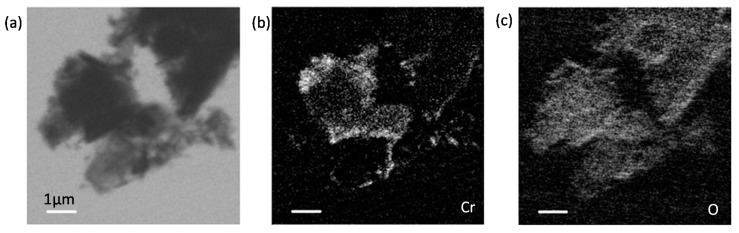
(**a**) Scanning transmission X-ray microscopy (STXM) image of GGBS-CH-S3 taken at 560 eV; (**b**) image contrast map of Cr; and (**c**) O for GGBS-CH-S3.

**Figure 8:**
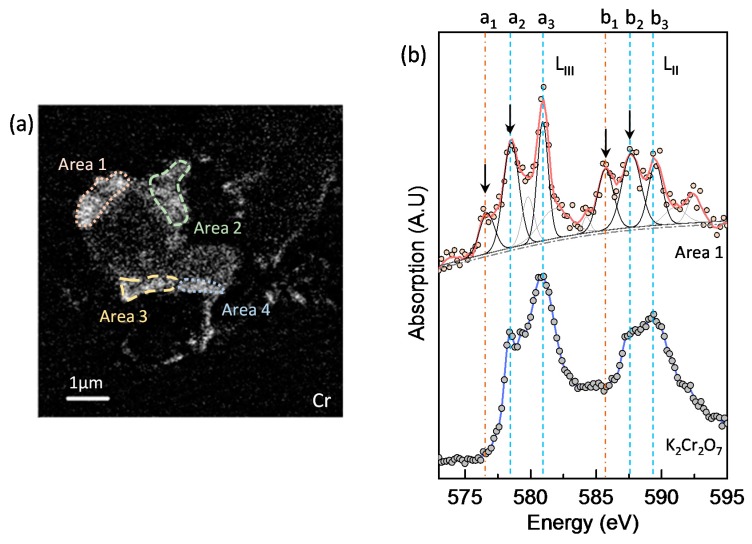
(**a**) Illustration of the analyzed areas in GGBS-CH-S3 for the Cr *L*_2,3_-edge NEXAFS in the Cr image contrast map (Figure 7b); (**b**) Cr *L*_2,3_-edge NEXAFS spectra for Area 1 in (**a**) and spectra from K_2_Cr_2_O_7_.

Figure 7 presents the X-ray absorption image of the GGBS-CH-S3 sample taken below Cr *L_3_*-edge at a photon energy of 560 eV, and the image contrast maps of Cr and O. Cr appeared to be localized at the edge of the GGBS particles, whereas O was equally distributed in the GGBS. In the Cr image contrast map, Area 1, as illustrated in Figure 8a, was selected for the Cr *L*_2,3_-edge NEXAFS analysis. We chose pure K_2_Cr_2_O_7_ as a reference for Cr(VI) spectra and used a series of Gaussian peaks to fit the spectra. In Figure 8b, K_2_Cr_2_O_7_ exhibits four distinct X-ray absorption features in the Cr *L*_2,3_-edge spectra, indicated as a_2_ (~578.5 eV), a_3_ (~580.9 eV), b_2_ (~587.7 eV), and b_3_ (~589.5 eV), where the peaks defined as a_3_ and b_2_ are induced, respectively, by the Cr *L_3_*, and *L_2_* absorptions. The additional peaks a_2_ and b_2_ result from the crystal field produced by the ligands with different electronegativities, which are non-linearly related to the magnitude of the crystal-field parameter (10Dq) [33,34]. Since the absorption shoulder features of Cr(VI) on the low energy side are very close to Cr(III)’s main absorption [32], the absorption peaks of a_2_ and b_2_ are overlapping contributions from both Cr(VI) and Cr(III). The low energy side features a_1_ and b_1_ (the arrows in Figure 8b) solely correspond to the octahedrally coordinated Cr(III), which is in excellent agreement with the chromite (FeCr_2_O_4_) spectrum and Cr_2_O_3_ [32,35,36]. The coexistence of Cr as both Cr(VI) and Cr(III) is presumably due to the superposition of the partially-reduced Cr(VI) and the physically adsorbed Cr(VI) onto the Ca(OH)_2_-activated GGBS. The remaining areas illustrated in Figure 8a show the same trends as Area 1, i.e., the coexistence of Cr(III) and Cr(VI). However, due to the lower Cr concentration, the areas have lower X-ray absorption intensities and a higher background noise.

**Figure 9:**
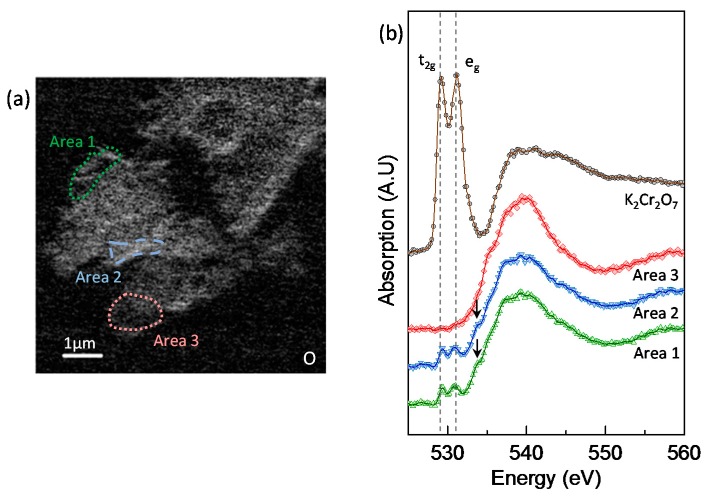
(**a**) Illustration of the analyzed areas in GGBS-CH-S3 for the O *K*-edge NEXAFS; (**b**) O *K*-edge NEXAFS spectra for each area in (**a**) and for K_2_Cr_2_O_7_.

The O *K*-edge NEXAFS analysis provided further evidence for the coexistence of Cr(VI) and Cr(III) on the Ca(OH)_2_-activated GGBS (GGBS-CH-S3), as shown in Figure 9. The O *K*-edge NEXAFS spectrum of the K_2_Cr_2_O_7_ exhibited intense pre-edge X-ray absorption peaks at 529.2 and 531.2 eV, and these can be attributed to the electronic excitation from the O 1s core level into the unoccupied t_2g_ and e_g_ molecular orbitals, respectively [37]. The O *K*-edge NEXAFS spectrum of pure Ca(OH)_2_ differs from the absorption features of CrO_4_^2−^ [24]; in this study, the presence of crystalline Ca(OH)_2_ was not observed in the entire area of the specimen. Area 1 and Area 2 appeared to contain two pre-edge, low-intensity absorption peaks arising from CrO_4_^2−^. In addition to the peaks, the features indicated by arrows at ~533 eV in the Area 1 and Area 2 spectra correspond to the O *K*-edge absorption peak of Cr(III)-oxyhydroxide, which has previously been observed in the spectrum of pyrite (FeS_2_) reacting with Cr(VI) [35]. Area 3 did not exhibit any CrO_4_^2−^ or Cr(III)-oxyhydroxide features because of the absence of any Cr in an oxidation state, as observed in Figure 7b.

### 3.6. Cr(VI) Dissolution and the Effects of Accelerated Carbonation

**Figure 10:**
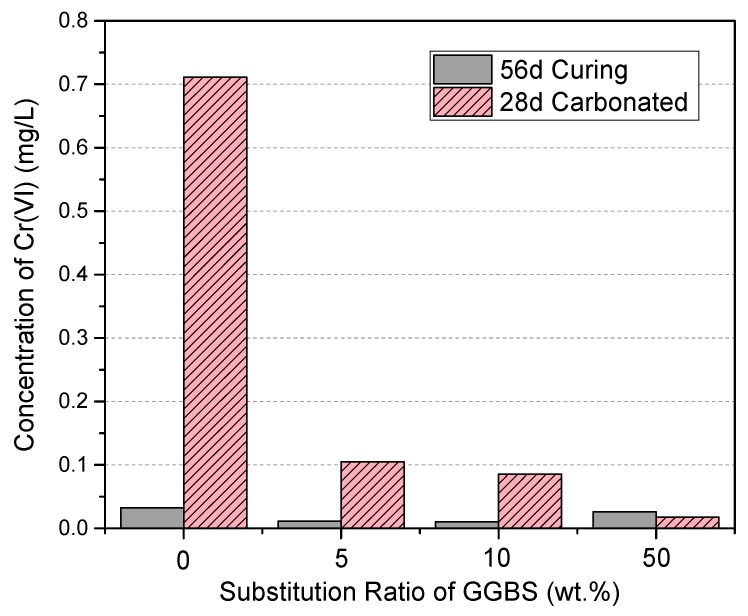
Tank leaching test results for the GGBS-PC paste before and after an accelerated carbonation test.

A comparison of the dissolved Cr(VI) concentration from the specimens before and after accelerated carbonation is shown in Figure 10. At 56 days, all the specimens that had not been exposed to atmospheric CO_2_ showed that relatively minimal amounts of Cr(VI) (i.e., less than the detection limit of 0.03 mg/L) were released from the GGBS-PC paste, regardless of the replacement ratio. Conversely, in the accelerated carbonation samples the dissolution of Cr(VI) depended strongly on the GGBS replacement ratio for PC. In pure PC, 0.7 mg/L of Cr(VI) was leached, whereas only 0.1 mg/L of Cr(VI) was leached from the GGBS-PC paste with a 5 wt % GGBS replacement. The Cr(VI) leached from the PC paste was the sum of the pre-added Cr(VI) (0.5 mg/L) and additional Cr(VI) (~0.2 mg/L) contained in the PC clinker, as already observed in the dissolution experiment (Figure 1b). For a sample with 50% of GGBS-replaced PC paste, we measure a very small amount of Cr(VI) below the detection limit of 0.03 mg/L. Pore solutions extracted from PC paste generally possess a pH in the range of 12.4 to 13.5 [38]. Our work revealed that the alkalinity resulting from the naturally formed Ca(OH)_2_ in PC hydration was sufficient for GGBS activation and enhanced GGBS’s capacity to reduce and immobilize Cr(VI), as observed in the solution samples (Figure 1b and Figure 2). Consequently, in a realistic PC paste, replacing PC with a minimal amount of GGBS had a significant impact on Cr(VI) reduction and immobilization, even with a 5 wt % replacement.

**Figure 11:**
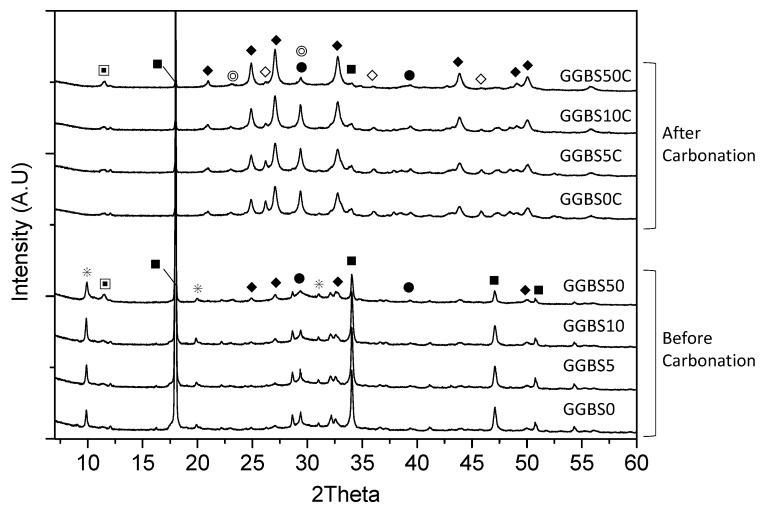
XRD patterns of the GGBS-PC paste with GGBS replacement ratios of 0, 5, 10, and 50 wt % before and after 28 days of accelerated carbonation. ◆: Vaterite, ♢: Aragonite, ■: Ca(OH)_2_, ◎: Calcite, ●: C-S-H, ✳: Monosulfate, and ⎕: Hydrotalcite.

Figure 11 shows the XRD patterns for the GGBS-PC paste, with different GGBS replacement ratios and either with or without carbonation. Peaks arising from monosulfate, Ca(OH)_2_, and C-S-H were detected for all GGBS-PC pastes without carbonation. For the 50 wt % GGBS-PC paste, we noted very weak peaks due to the presence of hydrotalcite (Mg_6_Al_2_(CO_3_(OH)_16_·4H_2_O)) and vaterite. This result indicated that replacing GGBS in PC increases the hydration products’ susceptibility to carbonation due to the reaction with atmospheric CO_2_. Previous studies have also confirmed that carbonation can change the PC paste’s porosity and that this can also affect the hardened specimen’s CO_2_ permeability [39,40].

Monosulfate diffraction patterns were not detected in all samples with carbonation, which suggests that complete monosulfate decomposition occurred in the carbonated samples. In addition, we detected the presence of CaCO_3_ polymorphs such as vaterite, aragonite, and calcite; these were the main, crystalline, accelerated carbonation products for all the specimens. In particular, as the carbonation progressed, the peak intensities of the CaCO_3_ polymorphs became stronger, as has been previously found [41]. Presumably, the formation of aragonite is linked to the carbonated C-S-H formed during PC clinker hydration, as has been observed in the young post-carbonation tricalcium silicate paste [42]. In general, Ca(OH)_2_ and C-S-H are the main hydration products involved in the carbonation of PC-based systems [43]; the carbonation occurs preferentially on Ca(OH)_2_, and precipitating CaCO_3_ polymorphs in the system [44,45]. However, carbonation in PC systems that contain 75% amounts of GGBS mainly occurs on the C-S-H phases, which results in vaterite formation [46]. C-S-H formed in GGBS-PC systems has a generally low Ca/Si ratio because of the Ca(OH)_2_ consumption compared to that of pure PC systems [47,48,49,50]. When C-S-H with a low Ca/Si ratio is exposed to CO_2_, both vaterite and aragonite, which are CaCO_3_ polymorphs, preferentially precipitate [51]. In the present study, after carbonation, C-S-H exhibited a peak intensity in GGBS50C that was lower (2θ = ~29°) than for those contained less GGBS. Furthermore, we observed that the more GGBS there was, the higher was the vaterite-related peak, which formed due to the carbonation of C-S-H with a low Ca/Si ratio.

**Figure 12:**
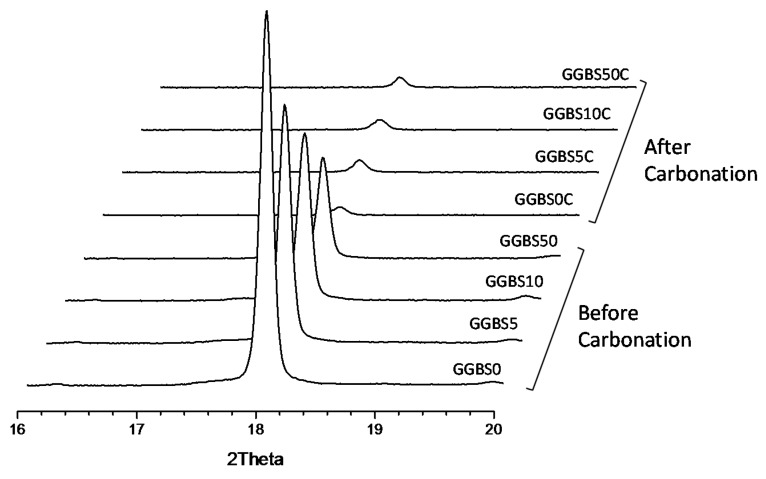
Comparison of the 001 basal reflection from Ca(OH)_2_ in GGBS-PC pastes before and after an accelerated carbonation. The numbers followed by GGBS indicate the replacement ratios of GGBS for PC.

Figure 12 shows the XRD peak intensity of the 001 basal reflection from Ca(OH)_2_ for the GGBS-PC pastes before and after carbonation. We observed a very intense peak for crystalline Ca(OH)_2_ in the pre-carbonation pure PC paste (GGBS0). Like anticipated, as GGBS’s replacement ratio in PC increased, Ca(OH)_2_’s peak intensity decreased due to the dilution of PC and to the consumption of Ca(OH)_2_ by GGBS. After carbonation, more Ca(OH)_2_ decreased in the pure PC paste (GGBS0) than Ca(OH)_2_ in the GGBS-PC paste. As a result, all the specimens exhibited similar Ca(OH)_2_ peak intensities, which supported the findings that C-S-H is more readily carbonated than Ca(OH)_2_ in GGBS-PC paste because the carbonation of C-S-H depends on the initial Ca(OH)_2_ amount present prior to carbonation [46]. Moreover, it can be demonstrated that the sufficient Ca(OH)_2_ in pure PC paste can buffer C-S-H carbonation. Presumably, the lower Cr(VI) dissolution in the carbonated GGBS-PC resulted from a pre-carbonation reductive process caused by dissolved anions such as S^2−^ (which are continuously released from GGBS during hydration) because S^2−^ can inhibit the oxidization of Cr(III) to Cr(VI) [18]. It should be noted that we do not have the direct evidence we need to identify the exact surface-promoted reductive transformation of Cr(VI) to Cr(III). It is likely that S^2−^ released during the hydration reaction is responsible for the reducing reactions in solution and that the reduced Cr species are physisorbed to hydrated materials. Equally, we cannot rule out the possibility that physisorbed S^2−^ located on hydrated-materials surfaces act as reducing agents for Cr reductive transformations. Further detailed studies are needed to distinguish the two different reaction pathways. Moreover, C-S-H formed in the GGBS-substituted PC system, which has a longer mean chain length [52], resulting in a larger surface area that can offer better surface adsorption, inclusion, and physical entrapment of Cr(VI). Based on the XRD and Cr(VI) dissolution results before and after carbonation, we concluded that partially replacing PC with GGBS can greatly reduce and immobilize Cr(VI) via dissolved anions into the pore solutions and via physical adsorption onto C-S-H. Even though C-S-H and hydration products are more susceptible to carbonation in the GGBS-PC system than in the pure PC system, Cr(VI) was not readily leached from the GGBS-PC paste after carbonation.

## 4. Conclusions

We found that without alkali activation GGBS is limited in its capacity to reduce Cr(VI). In contrast, the Ca(OH)_2_-activated GGBS demonstrated a considerably greater capacity for reducing and immobilizing Cr(VI). Therefore, we conclude that alkalinity is one of the dominating factors in determining the extent and mechanisms of reductive Cr(VI) dissolution and uptake reactions for GGBS. Furthermore, we found experimental evidence that Cr(VI) removal from the solution was due to its reactions with the dissolved anions, such as S^2−^, resulting from GGBS being under high alkaline conditions. X-ray absorption spectroscopy data identified the coexistence of Cr(III) and Cr(VI) on the Ca(OH)_2_-activated GGBS. This partial reduction of Cr(VI) to Cr(III) on the surface of GGBS suggests the presence of a surface-site-activated reductive mechanism. The Cr(VI) we observed on GGBS like results from physically adsorbed Cr(VI) on hydration products.

Our accelerated-carbonation experimental data indicate that the hydration products formed in the GGBS-PC paste, such as C-S-H, possessed a greater resistance to Cr(VI) leaching than those formed in pure PC. Our study suggests that even the used of 5% weight-based partial GGBS replacement for PC-based materials would be a suitable method for reducing and immobilizing Cr(VI) without violating the mechanical and physical integrity of PC [53]. We hypothesize that Ca(OH)_2_ generated during the early stage of PC hydration reactions promotes the dissolution and activation of GGBS, which results in an enhanced Cr(VI) reduction. It should be noted that observing S^2−^ concentrations in the dissolved solution will provide useful insights for determining and identifying further detailed reaction pathways. Future studies that use additional information to research similar experimental conditions are necessary to identify Cr(VI)’s exact reductive transformation pathways in hydrated cementitious materials. In addition, in order to understand the Cr(VI) reductive mechanism in GGBS, further detailed mechanistic and molecular research is needed to identify the exact state of physisorbed Cr(VI) on GGBS’s hydration products, how different types and amounts of GGBS alkaline activators influence Cr(VI) reduction and immobilization, and the factors that control Cr(VI) reduction pathways in carbonated GGBS-PC materials.

## Figures and Tables

**Table 1:** The chemical compositions of the raw materials.

Materials	Chemical Compositions (%)											
	SiO_2_	Al_2_O_3_	Fe_2_O_3_	CaO	MgO	SO_3_	Na_2_O	K_2_O	TiO_2_	P_2_O_5_	MnO	Total
PC	20.94	5.45	2.83	64.96	1.5	2.05	0.32	0.48	0.27	0.31	0.08	99.22
GGBS	33.03	14.82	0.42	41.44	6.51	2.07	0.27	0.27	0.53	0.04	0.18	99.9

**Table 2:** The mix proportions of the specimens.

Sample	Mix Proportions					Measurements
	Distilled Water (L)	GGBS (g)	CH (g)	PC (g)	Cr(VI) Concentration (mg/L)	
PC-S1	2	-	-	40	0.5	UV-vis, XRD, pH
GGBS-S1		40	-	-		UV-vis, XRD, pH
GGBS-CH-S1		40	20	-		UV-vis, XRD, pH, Soft X-ray absorption microscopy
PC-S2	2.4	-	-	48	0.5	UV-vis
GGBS-S2		48	-	-		
GGBS-CH-S2		48	24	-		
GGBS-CH-S3	2	40	20	-	0.5~100	UV-vis, XRD
GGBS-S4	2	40	-	-	-	Ion chromatography
GGBS-CH-S4	2	40	20	-	-	

GGBS = Granulated ground blast-furnace slag; CH = Ca(OH)_2_; PC = Portland cement; XRD = X-ray diffraction; UV-vis = Ultraviolet-visible spectrophotometry.
